# Supramolecular Photodimerization of Coumarins

**DOI:** 10.3390/molecules17021408

**Published:** 2012-02-03

**Authors:** Koichi Tanaka

**Affiliations:** Department of Chemistry and Materials Engineering, Faculty of Chemistry, Materials and Bioengineering, Kansai University, Suita, Osaka 564-8680, Japan; Email: ktanaka@kansai-u.ac.jp; Tel.: +81-06-6368-0861; Fax: +81-06-6339-4026

**Keywords:** photodimerization, coumarin, stereoselective reaction, supramolecular system

## Abstract

Stereoselective photodimerization of coumarin and its derivatives in supra-molecular systems is reviewed. The enantioselective photodimerization of coumarin and thiocoumarin in inclusion crystals with optically active host compounds is also described.

## 1. Introduction

**Scheme 1:**
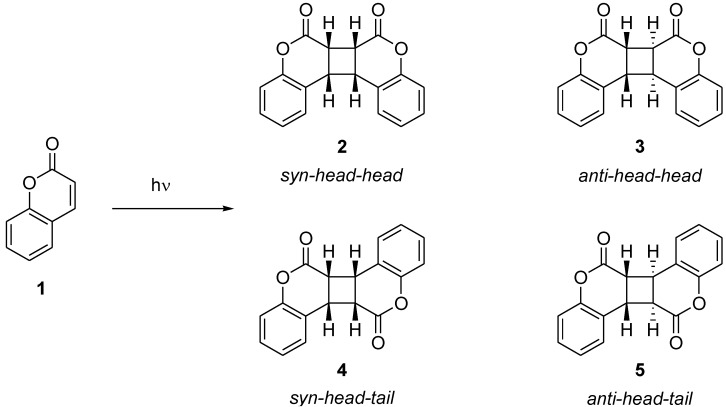
Photodimerization of coumarin.

**Scheme 2:**
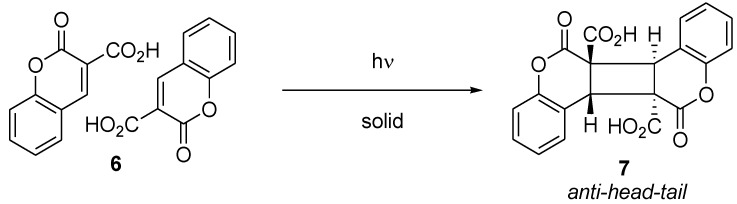
Photodimerization of coumarin-3-carboxylic acid in the solid state.

Photodimerization of coumarin and its derivatives has been studied extensively [1,2,3,4,5]. However, it is usually difficult to control the regio- and stereoselective [2+2] photodimerization of coumarins both in solution and in the solid state (Scheme 1). For example, the direct photoirradiation of coumarin in benzene afforded a mixture of *syn-head-head* **2**, *anti-head-head* **3**, *syn-head-tail* **4** and *anti-head-tail* **5** in the ratio 2.3:91.2:2.3:4.2, albeit in a low conversion of only 9% [4]. In contrast, photodimerization in 1,2-ethanediol gave a mixture of **2**, **3**, **4** and **5** in the ratio 59:22:19:0 in higher conversion (39%). The product ratio is also influenced by the multiplicity of the excited states involved [5]. Photoreactions in the solid state are thought to require precise orientation and separation of the two reacting double bonds within the maximum separation distance of 4.2 Å, as postulated by Schmidt, and some successful examples have been reported. Ramamurthy and Venkatesan pioneered the solid state photochemistry of coumarins and they identified the AcO and Cl substituents are identified as useful crystal engineering groups [1]. More recently, when monomer pairs of coumarin-3-carboxylic acid (**6**) are arranged in crystals such that the C=C double bonds are related by an inversion center and separated by 3.632 Å, the [2+2] cycloaddition reaction was achieved upon irradiation in the solid state [6] (Scheme 2).

In recent years, the photodimerization reaction of coumarins was found to be efficiently controlled by confinement within a supramolecular host such as cucurbituril, a Pd-nanocage, β-cyclodextrin, a bis-urea macrocycle, (*S,S*)-1,6-di(*o*-chlorophenyl)-1,6-diphenylhexa-2,4-diyne-1,6-diol, and (*S,S*)-1,6-di(2,4-dimethylphenyl)-1,6-diphenylhexa-2,4-diyne-1,6-diol. The enantioselective photodimerization of coumarin and thiocoumarin was also successfully achieved in inclusion crystals with chiral host compounds—(*R,R*)-*trans*-bis(hydroxydiphenylmethyl)-2,2-dimethyl-1,3-dioxacyclopentane and (*R,R*)-*trans-*2,3-bis(hydroxydiphenylmethyl)-1,4-dioxaspiro[4.4]nonane. In this review, the use of supra-molecular hosts for the regio-, stereo- and enantioselective photodimerization of coumarin and its derivatives is described.

## 2. Supramolecular Photodimerization of Coumarins in Solution

### 2.1. Cucurbituril

**Scheme 3:**
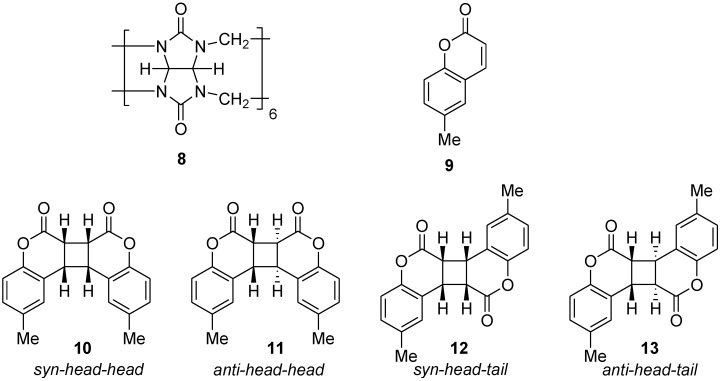
Photodimerization of 6-methylcoumarin.

Cucurbit[8]uril (**8**) has a cavity similar to that of cyclodextrins and has been shown to be an effective catalyst for the selective photodimerization of coumarins [7] (Scheme 3). The irradiation of 6-methylcoumarin (**9**) in water in the absence of **8** affords a mixture of four possible photodimers **10**-**13** in only 9% conversion. Conversely, in the presence of **8**, the reaction was found to be clean and efficient with exclusive formation of *syn* photodimers (**10** + **11**) as photoproducts. For example, the photodimerization of **9** in the presence of 50 mol% of **8** resulted in a 72% conversion to photodimers with a *syn*:*anti* ratio of >99:1.

### 2.2. Pd-Nanocages

**Scheme 4:**
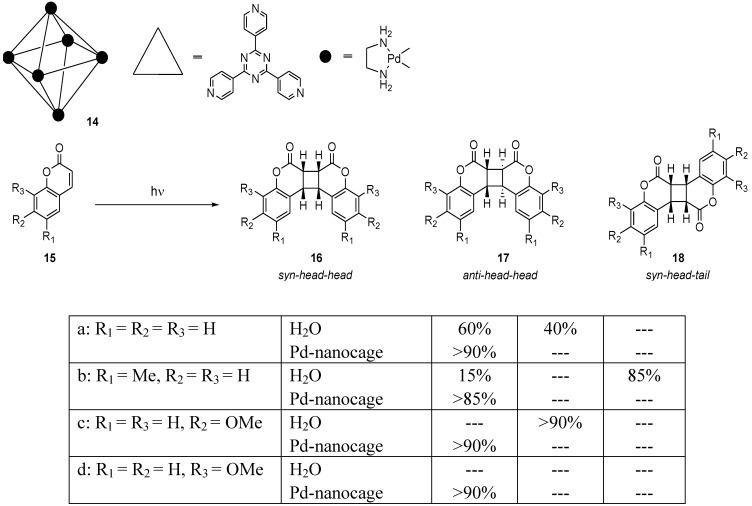
Photodimerization of coumarins within a Pd-nanocage and in H_2_O.

The irradiation of coumarin derivatives **15** within the Pd-nanocage **14 **selectively yields the *syn-head-head* dimers **16**, whereas in H_2_O, a mixture of dimers is obtained [8] (Scheme 4). For example, when irradiated as a host-guest complex, 8-methoxycoumarin (**15d**) exclusively formed the *syn-head-head* dimer **16d**, while **15d** itself was not sufficiently soluble to perform the photodimerization directly in water. The selective dimerization is interpreted to mean that coumarin monomers are preorganized by weak intermolecular interactions, such as hydrophobic, π-π, and CH-π interactions, between the host and guest to afford the *syn-head-head* dimer within the Pd-nanocage.

## 3. Supramolecular Photodimerization of Coumarins in the Solid State

### 3.1. β-Cyclodextrin

**Scheme 5:**
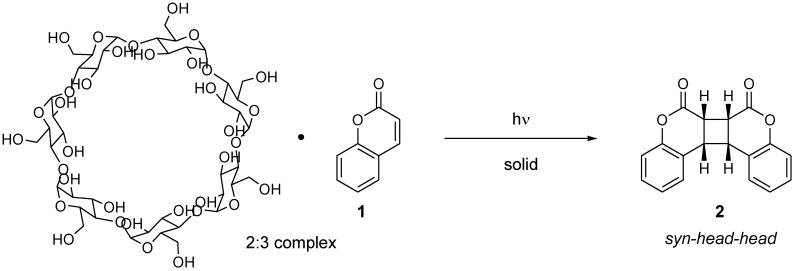
Photodimerization of coumarins in a β-cyclodextrin cavity.

The photodimerization of coumarin and eight of its derivatives was found to proceed selectively in crystalline inclusion complexes with β- and γ-cyclodextrins [9] (Scheme 5). For example, the irradiation of a 2:3 inclusion complex of β-cyclodextrin and coumarin in the solid state afforded the *syn-head-head* dimer in 64% yield, whereas its irradiation in water or as a neat solid provided **2** in low yield (~20%). The X-ray structural analysis of the 2:3 complex showed that the photodimerization likely occurs between coumarin molecules migrating inside a “reaction nano-tube” [10]. 

### 3.2. Bisurea Macrocycle

**Scheme 6:**
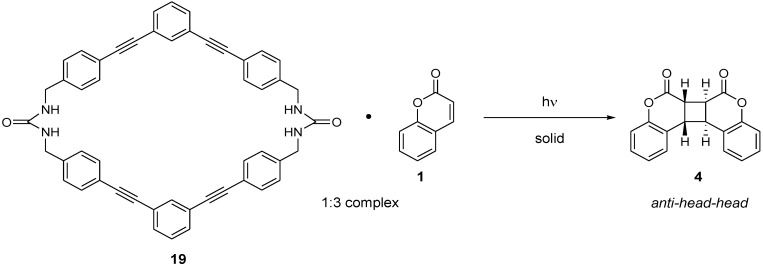
Photodimerization of coumarins in a bisurea macrocycle.

The bisurea macrocycle (**19**) with a diameter of ~9 Å forms a 1:3 inclusion complex with coumarin (**1**). The photoirradiation of the 1:3 complex for 96 h in the solid state under air afforded the *anti-head-head* dimer **2 **with 96% selectivity and 18% conversion. Interestingly, the reaction under N_2_ atmosphere gave a similar high selectivity (96%) with increased conversion (37%). The conversion could be further enhanced under Ar to 55% with no decrease in selectivity (97%) [11] (Scheme 6). 

### 3.3. Diacetylenediols

**Scheme 7:**
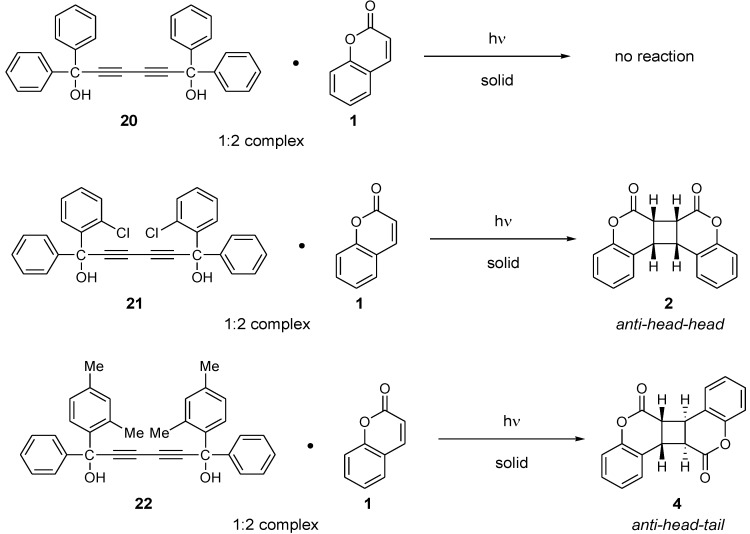
Photodimerization of coumarins inside inclusion crystals with diacetylenediols.

The photochemical behavior of coumarin on complexation with 1,1,6,6-tetraphenylhexa-2,4-diyne-1,6-diol (**20**), (*S,S*)-(−)-1,6-bis(o-chlorophenyl)-1,6-diphenyl-hexa-2,4-diyne-1,6-diol (**21**), and (*S,S*)-1,6-di(2,4-dimethylphenyl)-1,6-diphenylhexa-2,4-diyne-1,6-diol (**22**) has been investigated. It was observed that the inclusion complex of coumarin with the achiral host **20** was photoinert, whereas the complex with the chiral host **21** yielded the *syn-head-head* dimer in ca. 100% yield upon irradiation [12] (Scheme 7). X-ray structural analysis revealed that both the hydroxyl groups form OH-O hydrogen bonds with the carbonyl oxygen of the two coumarins and that the two double bonds are at an average distance of 3.8 Å. Contrary to this result, the irradiation of a 1:2 inclusion complex of the chiral host **22** with coumarin formed the *anti-head-head* dimer (±)-**4** in 94% yield [13]. 

## 4. Enantioselective Photodimerization of Coumarins in Crystalline Inclusion Complexes

**Scheme 8:**
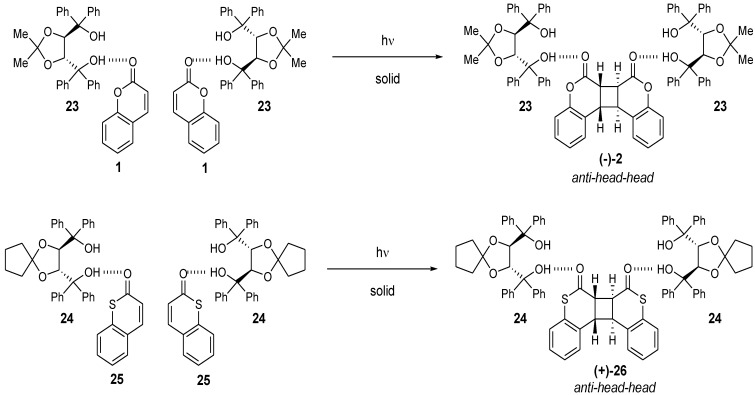
Single-crystal-to-single-crystalenantioselective photodimerization of coumarin in the inclusion crystals.

**Figure 1:**
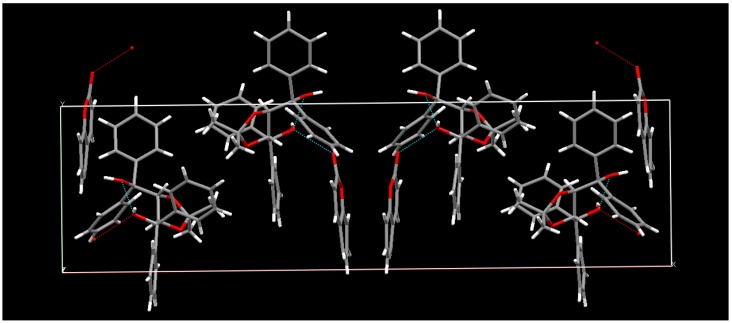
X-ray structure of the 1:1 inclusion complex of (−)-**23** and **1**.

**Figure 2:**
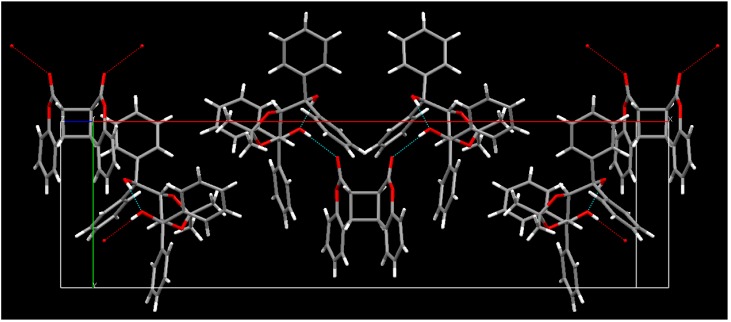
X-ray structure of the 2:1 complex of (−)-**23** and the optically active *anti-head-head* dimer (−)-**2**.

**Figure 3:**
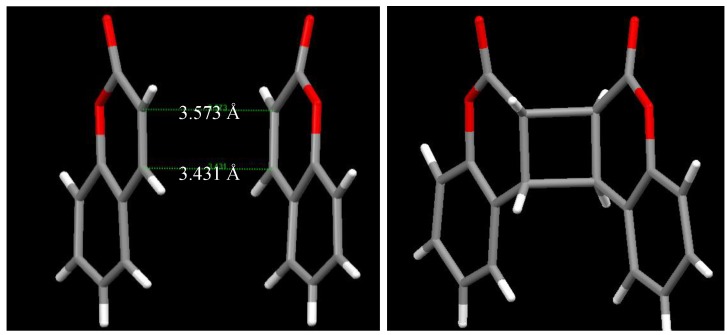
Mutual orientation of coumarin (left) and the *anti-head-head* dimer (−)-**2** (right) in the inclusion complex with (−)-**23**.

The single-crystal-to-single-crystal enantioselective photodimerization of **1** or thiocoumarin (**25**) was found to proceed efficiently in inclusion complexes with (*R,R*)-(−)-*trans*-bis(hydroxydiphenylmethyl)-2,2-dimethyl-1,3-dioxacyclopentane (**23**) or (*R,R*)-(−)-*trans*-2,3-bis(hydroxydiphenylmethyl)-1,4-dioxaspiro[4.4]nonane (**24**), respectively [14,15] (Scheme 8). Irradiation of the 1:1 inclusion complex of (−)-**23** and **1** in the solid state with a 400-W high-pressure Hg lamp (Pyrex filter, room temperature, 4 h) gave a 2:1 complex of (−)-**23** and the optically active *anti-head-head* dimer (−)-**2**. The *anti-head-head* dimer (−)-**2** was isolated in 99% yield and 100% *ee* by recrystallization of the 2:1 complex from DMF/H_2_O (5/1). Optically pure (+)-**2** was also obtained when the host compound (+)-**23** was used instead of (−)-**23**. The single-crystal-to-single-crystal nature and the steric course of the enantioselective photodimerization of **1** to the *anti-head-head* dimer (−)-**2** in the inclusion complex were investigated by X-ray crystallographic analysis. The results showed that two molecules of **1** were arranged by forming a hydrogen bond between the C=O of **1** and the O-H of **23** in the direction that gave the *anti-head-head* dimer (−)-**2** by photoirradiation and that the molecular aggregation had distances of 3.59 and 3.42 Å sufficiently short to readily permit topochemical reaction in the crystals. (Figure 1 and Figure 2) After photoirradiation, the bond distances of the cyclobutane ring were 1.6 and 1.57 Å, respectively (Figure 3). Recently, a new type of C2-symmetric bisphosphine ligand with a cyclobutane backbone has been synthesized starting from enantiopure (−)-**2** [16]. 

The photodimerization of **25** in the solid state gives a complex mixture of four possible dimers, although when irradiated in CH_2_Cl_2_, (±)-**26** is obtained [17]. The enantioselective photodimerization of **25** to the optically pure *anti-head-head* dimer (+)-**26** in the 1:1 inclusion complex of (−)**-24** with **25** was also found to proceed in a single-crystal-to-single-crystal manner [14,15]. The photoirradiation of the 1:1 inclusion complex of (−)-**24** with **25** in the solid state (400-W high-pressure Hg lamp, Pyrex filter, room temperature, 2 h) quantitatively gave a 2:1 complex of (−)**-24** with (+)**-26**; (+)**-26** was isolated in 73% yield and 100% ee by column chromatography. The single-crystal-to-single-crystal nature and the steric course of the photodimerization of **25** to the *anti-head-head* dimer (+)**-26** in the inclusion crystals were also investigated by X-ray crystallographic analysis. The two molecules of **25** in the 2:1 inclusion complex are related by a pseudo-twofold axis along the *c*-axis. The C=O of **25** and the O-H of (+)-**26** form a hydrogen bond in the direction that gives the *anti-head-head* dimer of (+)**-26**. The distances between the two ethylenic double bonds are sufficiently short (3.73 and 3.41 Å) to react easily and topochemically. After photoirradiation, the bond distances of the cyclobutane ring are both 1.60 Å. The crystal data are listed in Table 1. 

**Table 1:** Crystallographic data for a 1:1 complex of (−)-**23 **and **1**, a 2:1 complex of (−)-**23** with (−)-**2**, a 1:1 complex of (−)-**24** with **25**, and a 2:1 complex of (−)-**24** with (+)-**26**.

**Compound**	**(−)-23: 1**	**(−)-23: (−)-2**	**(−)-24: 25**	**(−)-24: (+)-26**
**Formula**	C_40_H_36_O_6_	C_40_H_36_O_6_	C_42_H_38_O_5_S_1_	C_42_H_38_O_5_S_1_
**Crystal system**	Monoclinic	Monoclinic	Monoclinic	Monoclinic
**Space group**	*C*2	*C*2	*P*2_1_	*P*2_1_
***a* (Å)**	35.59(4)	32.80(3)	10.235(2)	10.371(3)
***b* (Å)**	9.489(4)	9.467(3)	35.78(1)	34.70(2)
***c* (Å)**	10.03(1)	10.36(4)	9.422(2)	9.414(3)
***β* (°)**	102.70(4)	100.27(7)	91.00(2)	91.38(3)
***V* (Å^3^)**	3305(4)	3164(2)	3449(1)	3387(2)
***Z***	4	4	4	4
***D*_calc_**	1.23	1.29	1.26	1.28
***R***	0.101	0.114	0.065	0.078
**Temperature (°C)**	Room	Room	−50	−50

Recently, thermal [2+2] cycloaddition reactions of coumarin and thiocoumarin were found to occur in the above inclusion crystals [18]. Typically, coumarin and thiocoumarin are thermally unreactive for dimerization according to the Woodward-Hoffmann rules. Interestingly, however, the dimerization of coumarin occurred under high vacuum to form *anti-head-head* dimers in about 30% yield and 99% *ee*. For this reaction, both high-vacuum conditions and the presence of the host compound (−)-**23** were found to be essential.

## 5. Enantioselective Photodimerization of Coumarins in Solution

**Scheme 9:**
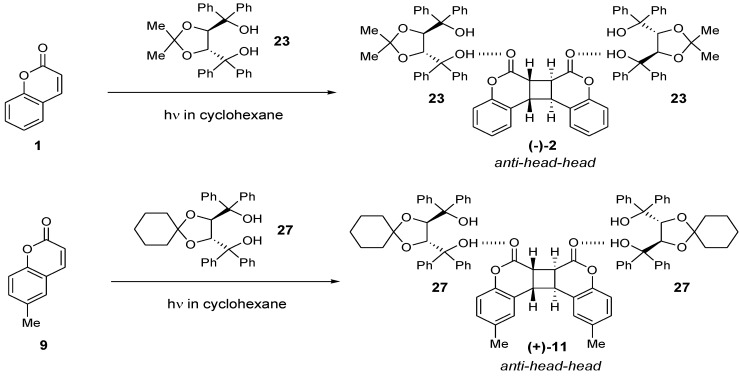
Photodimerization of coumarins in solution.

**Figure 4:**
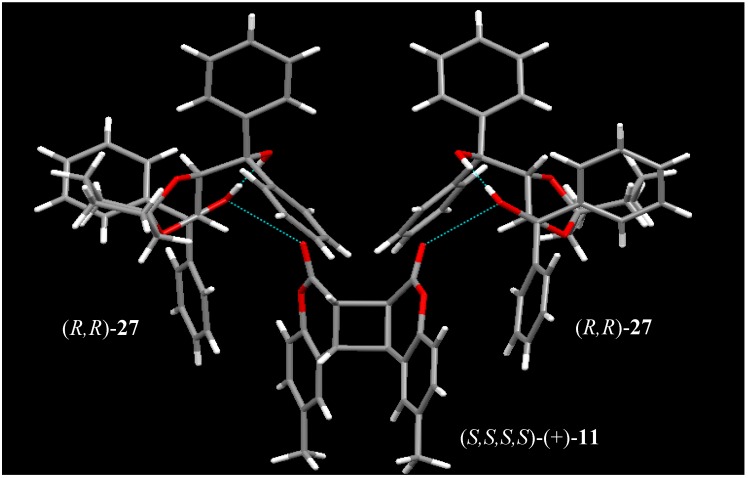
X-ray structure of the 2:1 complex of (−)-**27** and the optically active *anti-head-head* dimer (+)-**11**.

The enantioselective photodimerization reactions of **1** and **9** to the corresponding *anti-head-head* dimers (−)-**2** and (+)**-11** proceed efficiently in high enantioselectivity even in a homogeneoussolution in the presence of the optically active hosts (−)-**23**and (**−**)**-27**, respectively [19] (Scheme 9) (Figure 4). For example, a 2:1 inclusion complex of (*R*,*R*)**-**(−)**-27** and (*S*,*S*,*S*,*S*)-(+)-**11** was obtained in 60% yield and 95% *ee* upon photoirradiation of a cyclohexane solution of an equimolar mixture of (−)**-23** and **9**. Treatment of the inclusion complex with DMF/H_2_O gave (*S*,*S*,*S*,*S*)**-**(+)**-11** of >99% *ee* in 27% yield. One possible explanation of this reaction is as follows. The photodimerization of coumarin occurs in solvent but is reversible. In hydrocarbon and aromatic solvents, one enantiomer of the dimer complexes with the host, precipitates as inclusion crystals, and is protected from the reverse reaction. The other enantiomer is decomposed to the monomer by further irradiation in solution.
